# Green fluorescent protein-based monitoring of endoplasmic reticulum redox poise

**DOI:** 10.3389/fgene.2013.00108

**Published:** 2013-06-13

**Authors:** Julia Birk, Thomas Ramming, Alex Odermatt, Christian Appenzeller-Herzog

**Affiliations:** Division of Molecular & Systems Toxicology, Department of Pharmaceutical Sciences, University of BaselBasel, Switzerland

**Keywords:** endoplasmic reticulum, endoplasmic reticulum stress, glutathione, green fluorescent protein, hydrogen peroxide, unfolded protein response

## Abstract

Pathological endoplasmic reticulum (ER) stress is tightly linked to the accumulation of reactive oxidants, which can be both upstream and downstream of ER stress. Accordingly, detrimental intracellular stress signals are amplified through establishment of a vicious cycle. An increasing number of human diseases are characterized by tissue atrophy in response to ER stress and oxidative injury. Experimental monitoring of stress-induced, time-resolved changes in ER reduction-oxidation (redox) states is therefore important. Organelle-specific examination of redox changes has been facilitated by the advent of genetically encoded, fluorescent probes, which can be targeted to different subcellular locations by means of specific amino acid extensions. These probes include redox-sensitive green fluorescent proteins (roGFPs) and the yellow fluorescent protein-based redox biosensor HyPer. In the case of roGFPs, variants with known specificity toward defined redox couples are now available. Here, we review the experimental framework to measure ER redox changes using ER-targeted fluorescent biosensors. Advantages and drawbacks of plate-reader and microscopy-based measurements are discussed, and the power of these techniques demonstrated in the context of selected cell culture models for ER stress.

## Introduction

The largest endomembrane compartment in the eukaryotic cytoplasm, the endoplasmic reticulum (ER), has attracted increasing research interest over the past two decades (Schuldiner and Schwappach, 2013). The reason for this appears not to be the ER's long recognized function as the “founding organelle” of the secretory pathway, which involves the co-translational folding and post-translational processing of native polypeptide chains destined for cellular membranes or for secretion (Gidalevitz et al., 2013). The proliferation of ER-centered research is mostly due to the discovery of the signaling pathways of the unfolded protein response (UPR), which emanate from the ER in response to diverse protein folding stresses (Cox et al., 1993; Mori et al., 1993; Hetz, 2012). Indeed, the ER is now being recognized as a multifaceted signaling station with vital links to other cellular communication networks (Zhang and Kaufman, 2008; Appenzeller-Herzog and Hall, 2012; Deegan et al., 2012; Claudio et al., 2013; Kiviluoto et al., 2013). Furthermore, the ER maintains physical contact sites to the plasma membrane and essentially every other cell organelle, new functions of which are constantly being discovered (Helle et al., 2013).

The physiological outputs of the UPR are diverse and almost certainly dependent on cell type and the nature of the triggering stress. In general, they are thought to promote pro-survival mechanisms, until—upon prolonged and severe stress—the UPR switches to a network of signals culminating in the execution of the intrinsic, mitochondria-dependent apoptosis pathway (Hetz, 2012). Moreover, in most, if not all, contexts, UPR signaling is accompanied by the accumulation of intracellular oxidants (including reactive oxygen species, ROS), which is commonly referred to as oxidative stress and contributes to the detrimental outcome of exaggerated ER stress (Malhotra and Kaufman, 2007; Santos et al., 2009). As ROS also challenge protein homeostasis in the ER and, therefore, constitute an upstream trigger of ER stress (Buytaert et al., 2006; Malhotra and Kaufman, 2007; Santos et al., 2009), a vicious cycle can develop. This cycle is a critical element in the pathogenesis of various protein folding disorders, e.g. in the central nervous system (Matus et al., 2011). A key molecule counteracting reduction-oxidation (redox) imbalance in the stressed ER is the endogenous tripeptide glutathione (GSH) (Jessop and Bulleid, 2004; Molteni et al., 2004). By means of its cysteinyl side chain, GSH provides critical reducing power for catalyzed or non-catalyzed neutralization of oxidants, which, in the majority of reactions, results in generation of the dimeric, oxidized form of GSH, glutathione disulfide (GSSG) (Appenzeller-Herzog, 2011). Consequently, the manipulation of cellular GSH leads to UPR activation in response to ER hyperoxidation (in case of GSH depletion) (Cuozzo and Kaiser, 1999; Hansen et al., 2012) or ER hypooxidation (in case of GSH overload) (Kumar et al., 2011).

In spite of the established relationship between ER stress and oxidative stress, the origin of ER-stress-induced ROS is still being debated (Appenzeller-Herzog, 2011). For improved mechanistic understanding of underlying oxidative insults, tools for the specific quantification of ER redox conditions are therefore required. It is important to note though that the ER harbors so many redox couples (most of which are not in equilibrium with each other) that the precise definition of “ER redox conditions” is not possible (Appenzeller-Herzog, 2012). Nevertheless, the status of the GSH–GSSG redox couple is probably a vital measure for the thiol-disulfide homeostasis in the ER—a parameter critical for the formation of native disulfide bonds in substrate proteins and for the prevention of stress. Recently, the electrochemical reduction potential of GSH–GSSG (E_GSH_) in the ER was measured as −208 mV (Birk et al., 2013), confirming that the impact of GSH on the ER is fairly reducing. This was achieved using a glutaredoxin-fused redox-sensitive green fluorescent protein (roGFP) sensor (Hanson et al., 2004; Gutscher et al., 2008; Lohman and Remington, 2008), which was targeted to the ER of HeLa cells. Previous real-time measurements of redox changes in the mammalian ER were performed using GFP-based probes without defined specificity (van Lith et al., 2011; Kolossov et al., 2012). Another interesting redox-active molecule in the ER is the ROS hydrogen peroxide (H_2_O_2_), as it can diffuse through the ER membrane and thereby directly transmit a possible redox imbalance in the ER to other cell compartments (Appenzeller-Herzog, 2011; Bertolotti et al., 2013). H_2_O_2_ is generated *in situ* by the action of ER-resident oxidases such as endoplasmic oxidoreductin 1α (Ero1α) (Ramming and Appenzeller-Herzog, 2012). Changes in [H_2_O_2_] in the ER can be visualized by monitoring the dithiol-disulfide state of the fluorescent protein-based probe HyPer (Belousov et al., 2006; Enyedi et al., 2010; Wu et al., 2010), although this readout is doubtlessly also influenced by the ER-resident machinery for disulfide-bond formation and is therefore not a *bona fide* measure for [H_2_O_2_] (Mehmeti et al., 2012; Ruddock, 2012).

In this paper, we outline detailed protocols for the assessment of ER redox conditions using targeted fluorescent protein sensors in either plate-reader- or microscopy-based fluorescence readouts. The suitability of each method for specific experimental problems is discussed.

## ER-targeted roGFP sensors

For the measurement of dynamic redox changes in the ER of mammalian cells, two codon-optimized roGFP sensors with suitable midpoint reduction potentials are now available: roGFP1-iE_ER_ and Grx1-roGFP1-iE_ER_ (Birk et al., 2013). The latter probe specifically reports E_GSH_ (Meyer and Dick, 2010; Birk et al., 2013). Oxidized roGFPs harbor a disulfide bond in the GFP beta-barrel, formation of which changes the excitation spectrum of the protein (see below). Thus, the dithiol-disulfide ratio of transiently transfected, ER-targeted roGFPs can be measured by fluorescence excitation analysis. An alternative, relatively easy approach is the trapping of the roGFP redox state by treatment of cells with a membrane-permeable alkylating agent followed by immunoprecipitation, SDS-PAGE, and immunoblotting where the oxidized fraction of roGFP is identified by its increased gel mobility (Birk et al., 2013). However, this approach yields a redox distribution, which is consistently more reduced than when assessed by means of fluorescence readouts (Birk et al., 2013) (Table 1). Accordingly, the two methods for fluorescence excitation analysis outlined in the following sections are preferable when precise quantification of the extent of oxidation of ER-targeted roGFP probes is desired.

**Table 1:** **OxD values (percentage ± s.d.) of roGFP1-iE_ER_ and Grx1-roGFP-iE_ER_ obtained by different methods**.

	**Western blot**	**Microscopy**	**Excitation spectra analysis**
roGFP1-iE_ER_	54% ± 6	81% ± 13	81% ± 7
Grx1-roGFP1-iE_ER_	70% ± 5	93% ± 2	90% ± 3

## Determination of the redox state of roGFP1-iE_ER_ in mammalian cells using a fluorescence plate reader

### Reagents

- HeLa cells (ATCC).
- Mammalian expression plasmid containing the genetically encoded roGFP sensor.
- FuGene HD (Promega).
- Dulbecco's modified Eagle medium (DMEM) high glucose (Sigma), containing 10% fetal bovine serum, 100 U/ml penicillin, 100 μg/ml streptomycin.
- Trypsin-EDTA.
- HEPES-buffer (20 mM Hepes, 130 mM NaCl, 5 mM KCl, 1 mM CaCl_2_, 1 mM MgCl_2_, 5.5 mM Glucose, pH 7.4).
- Dithiothreitol (DTT, AppliChem), 1 M Stock in HEPES-buffer (freshly prepared).
- Diamide (Sigma), 0.5 M stock in H_2_O, or 2,2′-dithiodipyridine (DPS, Sigma), 10 mM stock in H_2_O.

### Equipment

- Fluorescence plate reader equipped with a monochromator with an excitation wavelength range from 350 to 500 nm and an emission wavelength of 530 nm. A bottom-up reader should be used such as Gemini EM fluorescence microplate reader (Molecular Devices) with SoftMax Pro software.
- Clear, flat-bottomed 96 well plate.

### Procedure

#### Cell transfection

1. Trypsinize cells and seed them into two 60 mm dishes at a density of 9 × 10^4^ cells per dish.
2. Transfect cells with 4 μg of sensor plasmid using FuGene HD according to the manufacturer's recommendation. Researchers are encouraged to choose the optimal transfection reagent and protocol for their particular cell line.

Transient transfection can only be used when the transfection efficiency is high enough. For other cell types, it might be difficult to obtain sufficient transfection efficiency, necessitating the generation of a cell line stably expressing the sensor (see also below).

#### Cell preparation

**Figure 1:**
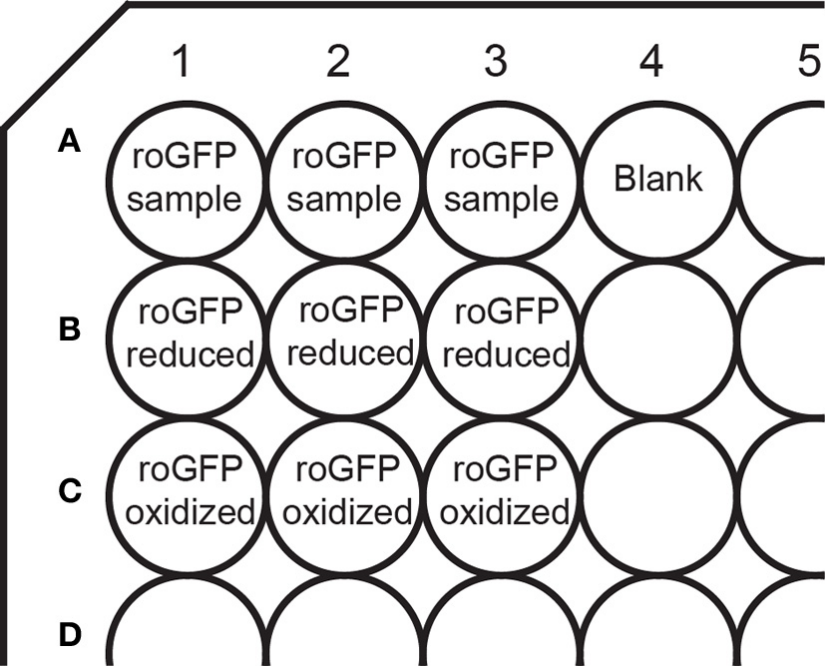
**Layout of a 96-well plate for measurements on a plate reader.** The plate contains wells for fully oxidized and fully reduced controls, as well as a blank well containing buffer only for blank subtraction.

1. Trypsinize cells 48 h post-transfection and resuspend in complete DMEM.
2. Spin 3 min at 200 × g and carefully aspirate medium.
3. Wash cell pellet with HEPES-buffer and spin again 3 min at 200 × g.
4. Aspirate HEPES buffer and resuspend cells in 1 ml HEPES buffer. Avoid harsh pipetting to prevent shearing of the cells.
5. Distribute the cells to 9 wells of a 96 well plate, (100 μl of cell suspension per well) as outlined in Figure 1.
6. Pipet 200 μl of HEPES buffer in an additional well (Figure 1) for blank control. The background fluorescence excitation spectra of buffer-filled wells and of wells containing untransfected cells in buffer were found to be identical (our unpublished data).
7. Spin plate 2 min at 200 × g.

#### Setting up the plate reader

1. Pre-warm the instrument to 37°C.
2. Choose “bottom read.”
3. Set the excitation spectrum from 350 to 500 nm with 5 nm intervals. Emission is detected at 530 nm with a cutoff at 515 nm.

#### Measurement

1. To the wells in the first lane (Figure 1) carefully add 100 μ l HEPES-buffer without disturbing the monolayer.
2. Prepare a 20 mM DTT solution in HEPES-buffer and carefully add 100 μ l to the wells in the second lane.
3. Incubate for 5 min.
4. Prepare a 10 mM diamide or a 1 mM DPS solution (from the respective stock solution) in HEPES-buffer and add 100 μ l to the wells of the third lane.
5. Start the measurement.

### Calculation of the degree of roGFP oxidation (OxD)

To obtain blank-corrected excitation spectra from untreated, reduced, and oxidized cells, triplicate emission intensity values at all excitation wavelengths are averaged, the corresponding blank values subtracted and plotted against the excitation wavelength. In the resulting spectra, the values at 390 and 465 nm are extracted to calculate OxD using the following equation:

$${\text{OxD}}_{\text{roGFP}}=\frac{R-R_{\text{red}}}{\frac{I_{390\text{ nm}}\text{ox}}{I_{390\text{ nm}}\text{red}}\left(R_{\text{ox}}-R\right)+\left(R-R_{\text{red}}\right)}$$

where *R*, *R*_red_, and *R*_ox_ represent the 390:465 nm fluorescence ratios at steady state, after complete reduction or oxidation, respectively. I390 nm is the average fluorescence emission at 390 nm excitation under oxidized or reduced conditions and serves as calibration factor. The obtained OxD value is expressed as percentage of sensor oxidation.

### Expected results

**Figure 2:**
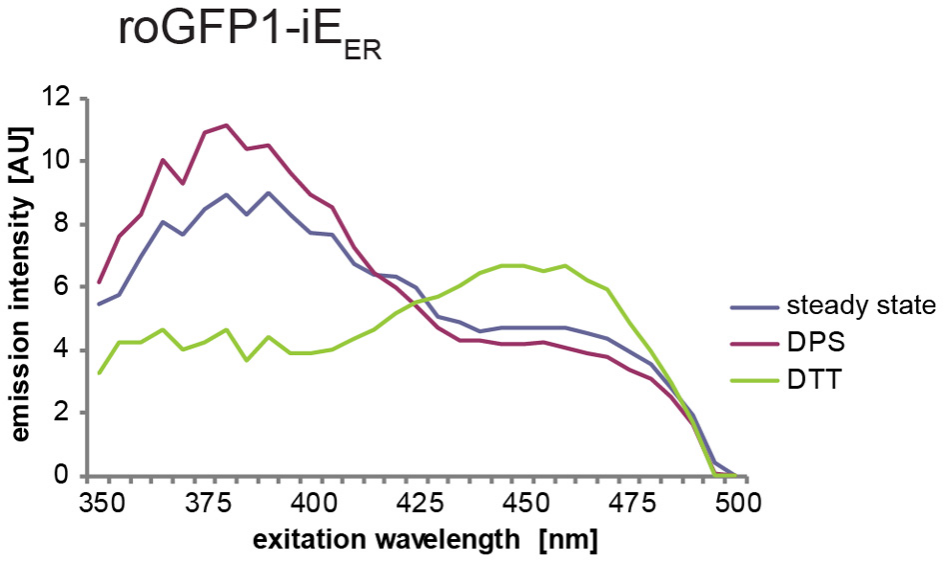
**Exemplary spectra of roGFP1-iE_ER_ transiently transfected into HeLa cells.** The lines in the graph represent the averaged and blank corrected fluorescence intensities from cells at steady state (blue), upon oxidation with DPS (red), or reduction with DTT (green). The redox-dependent ratiometric behavior of the two peaks of roGFP1-iE_ER_ at 390 and 465 nm is visible.

Depicted in Figure 2 is a typical redox state analysis of roGFP1-iE_ER_ carried out on a fluorescence plate reader. The peak values extracted from these spectra are to be used to calculate the OxD value as described above.

An advantage of this method is its high reproducibility, which allows reasonable estimates of the redox conditions in the ER. The fact that the measurement is done over a whole cell population comprising different expression levels of the sensor results in a robust readout. The 96-well format is useful for testing the influence of several conditions like, for example, different pharmacological compounds or knockdown of different genes on the same plate. Furthermore, the quality of the experiment and the sensor performance can readily be appraised by inspection of the three excitation spectra (Figure 2). However, the plate reader method is not suitable for live experiments where temporal resolution of the measurement under physiological conditions is of special interest. This holds true for the monitoring of both rapid redox changes, which are difficult to visualize in the plate-reader setup, and long-lasting redox trends, which requires analysis of adherent cells under preferably standard growth conditions.

## Dynamic and steady state roGFP1-iE_ER_ measurements in mammalian cells using ratiometric video microscopy

**Figure 3:**
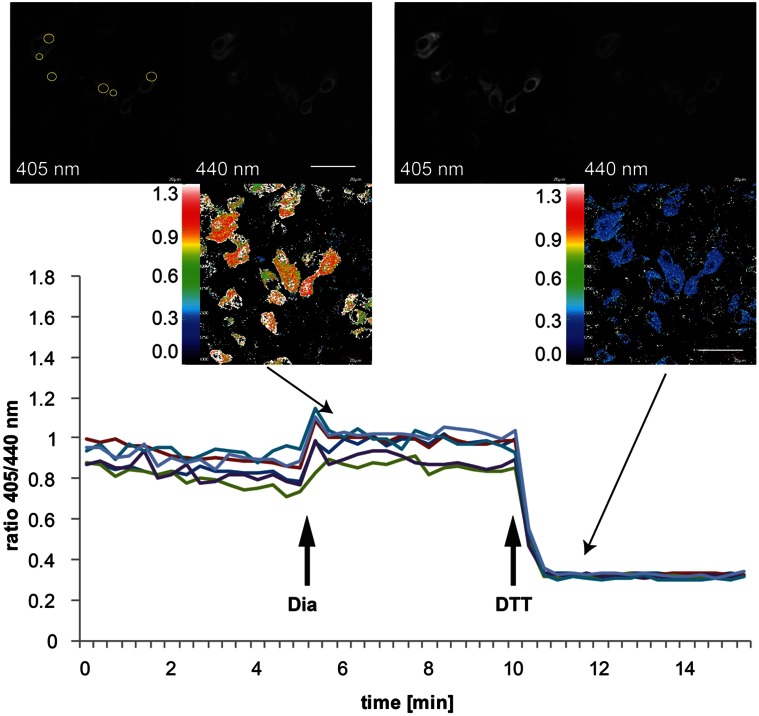
**Imaging of dynamic changes in the Grx1-roGFP1-iE_ER_ excitation ratio in transiently transfected HeLa cells.** HeLa cells were seeded to MatTek glass bottom dishes and transfected on the following day with Grx1-roGFP1-iE_ER_. Forty eight hours post-transfection, the cells were analyzed by ratiometric laser scanning microscopy. The dynamic changes of the ratios of emission intensities (405 nm excitation):(440 nm excitation) obtained from individual cells upon treatment with 5 mM diamide (Dia), followed by addition of 10 mM DTT are plotted against time. The images in the insets show exemplary fluorescence pictures in the 405 nm or 440 nm channel, respectively, as well as the corresponding ratiometric images. In the upper left micrograph, the positions of ROIs for ratiometric image analysis are indicated as yellow circles.

Live cell imaging can be used to obtain information about the redox state of roGFP1-iE_ER_ as well as to monitor dynamic changes thereof. Assessment of the steady-state ER redox environment is achieved by conducting a three point measurement consisting of a series of three pictures representing (1) the ground state, (2) the completely oxidized, and (3) the completely reduced state of the sensor. Figure 3 shows a real-time experiment where the three states of Grx1-roGFP1-iE_ER_ were induced and imaged by ratiometric video microscopy with the sequential addition of oxidant (diamide) and reductant (DTT) to the specimen. The OxD values obtained by this method are comparable with those obtained when using the fluorescence plate reader assay (Table 1) (Birk et al., 2013), which validates the data acquired under the less physiological conditions in the plate reader setup.

### Reagents

- HeLa cells (ATCC).
- Mammalian expression plasmid containing the genetically encoded roGFP sensor.
- FuGene HD (Promega).
- Dulbecco's modified Eagle medium (DMEM) high glucose (Sigma), containing 10% fetal bovine serum, 100 U/ml penicillin, 100 μg/ml streptomycin.
- Trypsin-EDTA.
- HEPES-buffer (see above) or DMEM without phenol red. Phenol red can be extracted from conventional DMEM by addition of 0.5 g charcoal per 50 ml of medium and incubation for 2 h under agitation. The charcoal is then removed by filtration. Alternatively, phenol red-free DMEM is commercially available.
- DTT, 1 M Stock in HEPES-buffer.
- Diamide, 0.5 M stock in H_2_O.

### Equipment

- Olympus Fluoview 1000 laser scanning confocal microscope equipped with a 60× oil immersion objective (NA 1.40), a 405 nm laser diode, and a 440 nm laser diode.
- Climate chamber with CO_2_ vent and humidifier.
- Automated focus control to correct for thermic drift.
- 35 mm dishes with glass bottom (MatTek Corporation).

### Procedure

#### Cell transfection

1. Trypsinize cells and seed to glass bottom dish at a density of 2 × 10^5^ cells per dish in 400 μl DMEM only onto the glass bottom in the middle of the plate.
2. Carefully add 1.6 ml DMEM on the following day.
3. Transfect cells with 2 μg of sensor plasmid using FuGene HD according to the manufacturer's recommendation.

#### Microscope settings

Based on the excitation maxima of roGFP1-iE_ER_ (see Figure 2), the 405 and 440 nm laser lines are used. The emission window is set to 500–600 nm. Images are acquired in sequential frame mode, separating the two channels. The scan speed is set to 8 μs/pixel. If available, use an automated focus control to correct for drift.

It is important to control the surrounding climate of the sample, especially when measurements are performed over a long period of time. Accordingly, the climate chamber needs to be closed, and usage of CO_2_ and a humidifier is advised. To facilitate addition of compounds like for example oxidants or reductants despite the closed lid, it is recommendable to install a feed pipe.

To prevent saturated signals, calibration of the microscope with cells cultured in separate wells and treated with reductant or oxidant, as explained by Morgan et al. (2011), is possible when using stably expressing cell lines. However, in our own experience, when using transiently transfected cells, such calibration is not feasible owing to varying expression levels between different cells and samples. We therefore routinely pre-estimate the maximal emission levels of the sensors (i.e., after addition of reductant or oxidant) in the two channels in order to determine suitable gain settings. In practice, this means that the sensitivity of the photomultiplier tube of the 440 nm channel is adjusted to a very low signal, since complete reduction of the sensor will strongly increase the fluorescence signal in this channel. However, since the photomultiplier of the 405 nm channel has to be adjusted equally, a setting is required in which the 440 nm channel emits the lowest signal possible, but the signal in the 405 nm channel is still visible. Furthermore, in order to be able to work with lower laser power, we recommend opening the pinhole completely. This will decrease the risk of light-induced artifactual oxidation. On the flip side, this measure lowers spatial resolution.

#### Measurement

1. Fill the humidifier with water and open the CO_2_ vault when performing a long-term measurement. For steady-state measurements, preheating of stage and objective are sufficient.
2. Set the temperature of the climate chamber to 41°C, preheat the lid to 44°C and the objective to 37°C.
3. Let the system equilibrate for one hour.
4. Wash the cell monolayer in the imaging dish two times with DMEM without phenol red. Steady-state measurements can also be performed in HEPES-buffer instead.
5. Add 1 ml medium without phenol red or HEPES-buffer to the cells and mount the dish with the probe onto the heated stage.
6. Let the system equilibrate for at least 15 min.
7. Use transmitted light to find an appropriate field for imaging.
8. *For steady state measurements:* set the time intervals to 5 min and take three pictures, one as starting point, one after addition of oxidant, and one after the addition of reductant (see below).
  *For dynamic long-term measurements:* choose an appropriate time interval between different frames (depending on the length of measurement and the kinetics of the redox response to be analyzed). Typically, for 3 h we recommend to use 10 min.
9. Adjust the photomultiplier tube of both channels to equal voltage following the guidelines discussed in section Microscope settings.
10. Start the measurement.
11. Add 10 mM diamide in 1 ml DMEM without phenol red or HEPES-buffer.
12. Add 60 mM DTT in 1 ml DMEM without phenol red or HEPES buffer on top.

#### Image analysis

1. Image analysis can be done using different programs including ImageJ (Morgan et al., 2011); we generally use the background subtraction and the series analysis tool of the FV-100 software (Olympus) installed on our microscope.
2. Set regions of interest (ROIs) within selected cells (Figure 3). Exclude cells with saturated pixels. We usually set one ROI per cell and analyze as many cells in the field of view as possible, at least 10. Especially when performing long-term measurements, it is recommendable to use relatively small ROIs, since the cells may move during image acquisition. Using a smaller ROI will minimize the risk of “loosing” the cell due to migration.
3. Export the fluorescence intensity data to Microsoft excel and use the formula provided in section “Calculation of the degree of roGFP oxidation (OxD)” for calculation of OxD values.
4. The ratio between the channels is calculated as emission intensity at 405 nm excitation divided by the emission intensity at 440 nm excitation.

### Live imaging of roGFP1-iE_ER_ in the stressed ER

**Figure 4:**
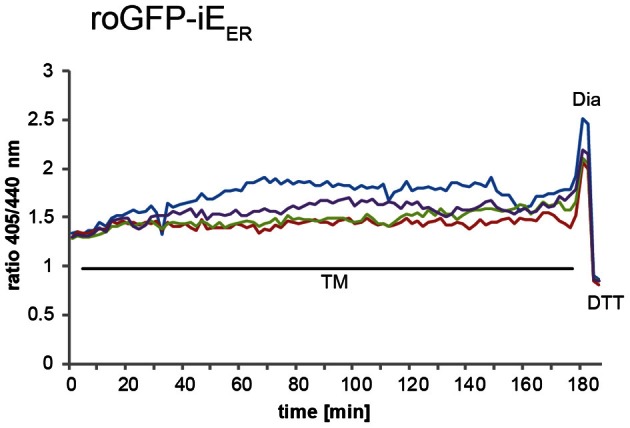
**Live imaging of roGFP1-iE_ER_ upon tunicamycin treatment.** HeLa cells transiently expressing roGFP1-iE_ER_ were analyzed by ratiometric laser scanning microscopy 48 h post-transfection. Where indicated, 1 μg/ml tunicamycin (TM), 0.5 mM diamide (Dia), and 20 mM DTT were added to the specimen. The dynamic ratio changes of individual cells were plotted against time.

Induction of ER stress can be achieved by treatment of cells with different compounds including DTT, which prevents disulfide-bond formation, thapsigargin, an inhibitor of the ER Ca^2+^ ATPase, or tunicamycin, an inhibitor of protein glycosylation. Induction of the full UPR upon application of these drugs to tissue culture cells typically occurs after 10–30 min (DuRose et al., 2006). Accordingly, possible effects of ER stress on the ER redox environment can readily be monitored by real-time video microscopy. As an example, Figure 4 shows, how live cell imaging can be used to assess the effect of tunicamycin treatment on the redox state of roGFP1-iE_ER_ in transiently transfected HeLa cells. Cells react heterogeneously to tunicamycin-induced ER stress. While many of them show no discernible changes in the ER redox environment, a subpopulation of cells display moderate hyperoxidation of roGFP1-iE_ER_ after a lag phase of ~100 minutes [compare also to the experiment published in Birk et al. (2013)].

## Determination of ER redox changes in flipin TRex 293 cells with doxycycline-inducible gene expression using HyPer_ER_

**Figure 5:**
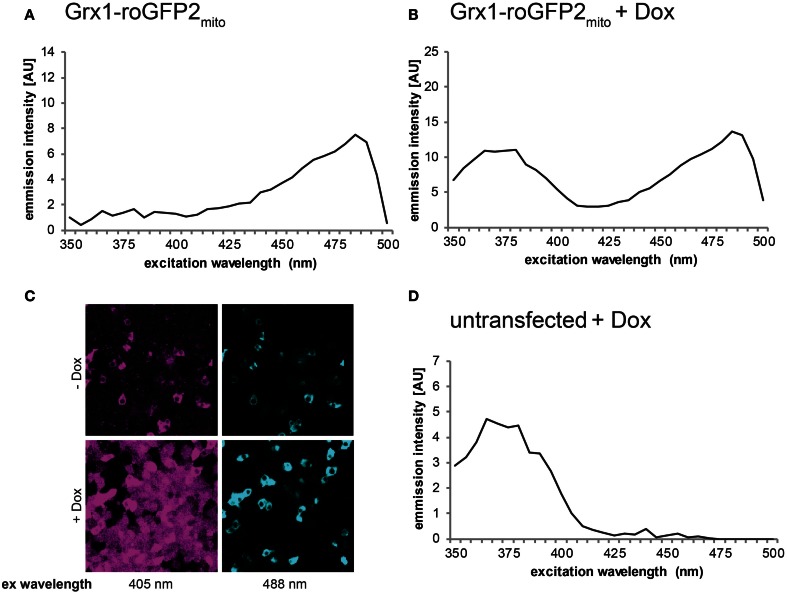
**Fluorescence of doxycycline prohibits roGFP-based redox analysis.** Cells were transiently transfected with mitochondrial Grx1-roGFP2 (Grx1-roGFP2_mito_) (Gutscher et al., 2008) and induced with doxycycline (Dox) for 24 h. While non-induced cells displayed a normal roGFP spectrum **(A)**, Dox treated cells showed an altered peak at 375 nm excitation **(B)**, which overlapped with the fluorescence produced by Dox alone when added to untransfected cells **(D)**. The same effect was observed using ratiometric laser scanning fluorescence microscopy, as evidenced by ubiquitous fluorescence upon excitation (ex) with the 405 nm laser line **(C)**.

Besides pharmacological induction, ER stress can also be a result of oxidative challenge. For instance, a recent study demonstrated UPR induction in response to doxycycline-induced expression of a hyperactive mutant (C104A/C131A) of Ero1α in FlipIn TRex 293 cells (Hansen et al., 2012). This ER-resident oxidase generates disulfide bonds, which entails the concomitant production of equimolar amounts of the reactive oxidant H_2_O_2_ (Ramming and Appenzeller-Herzog, 2012). Fluorescence-based readouts for characterization of the shift in the ER redox balance resulting from Ero1α-C104A/C131A induction, however, are not straightforward. Doxycycline displays a fluorescence excitation peak at around 375 nm, which significantly overlaps with the roGFP excitation spectrum (Figure 5) and cannot be eliminated by repeated washing of the cell monolayer (our unpublished data). To overcome this problem, the use of a different genetically encoded, ratiometric redox probe, HyPer (Belousov et al., 2006), shall be described in this section. In contrast to roGFP, the red-shifted excitation spectrum of HyPer does not overlap with the 375 nm peak of doxycycline and, hence, fluorescence spectrum analysis of HyPer is applicable in doxycycline-inducible cell systems. Alternatively, doxycycline-dependent changes in the redox state of roGFP-based probes can be visualized by an immunoprecipitation/Western blot approach, as has been published (Birk et al., 2013).

A peculiarity of the HyPer probe is its ability to rapidly and directly react with H_2_O_2_ (Belousov et al., 2006). This feature has led to the suggestion that HyPer is a specific H_2_O_2_ probe. While this may be true for cell compartments like the cytosol or the nucleus, which harbor potent disulfide-reducing machinery, the relatively oxidized redox state of HyPer in the environment of the ER (Enyedi et al., 2010; Wu et al., 2010; Malinouski et al., 2011) most likely does not exclusively reflect the presence of H_2_O_2_, but also of other oxidizing factors such as protein disulfide isomerases (Appenzeller-Herzog and Ellgaard, 2008; Mehmeti et al., 2012; Ruddock, 2012).

### Procedure

The protocol to determine the fluorescence excitation spectrum of HyPer in FlipIn TRex 293 cells is largely identical to the excitation spectrum analysis of roGFPs in HeLa cells (described in Section “Determination of the redox state of roGFP1-iE_ER_ in mammalian cells using a fluorescence plate reader”). Thus, only explicit differences between the two protocols will be discussed in this section.

- In contrast to HeLa cells, FlipIn TRex 293 cells require stable transfection of the redox probe to reach an acceptable signal-to-noise ratio for fluorescence measurements in a plate reader. Therefore, for the experiments described further below, FlipIn TRex 293:Ero1α-C104A/C131A cells (Hansen et al., 2012) were transfected with HyPer_ER_ (Enyedi et al., 2010) followed by clonal selection with G418.
- 7.5 × 10^5^ cells are seeded into 35 mm dishes (e.g., in 6-well plates) and the expression of the inducible cDNA initiated on the following day by addition of 1 μg/ml doxycycline (from a 1000× aqueous stock solution) 24 h ahead of analysis.
- In order to yield the fully oxidized form of HyPer_ER_ immediately prior to fluorescence scanning in the plate reader, cells in the 96-well plates are treated with 100 μM H_2_O_2_ instead of DPS or diamide. As the presence of 5 mM glucose in the cell resuspension buffer lowers the oxidizing effect of H_2_O_2_ (our unpublished data), presumably due to glucose-mediated reduction of H_2_O_2_, glucose needs to be omitted from the HEPES-buffer.
- The plate reader settings need to be adjusted to the excitation spectrum of HyPer. Thus, excitation wavelength ranges from 410 to 510 nm with 5 nm steps. Emission is detected at 535 nm with a cutoff at 530 nm.

### Expected results exemplified by conditional expression of Ero1α-C104A/C131A

**Figure 6:**
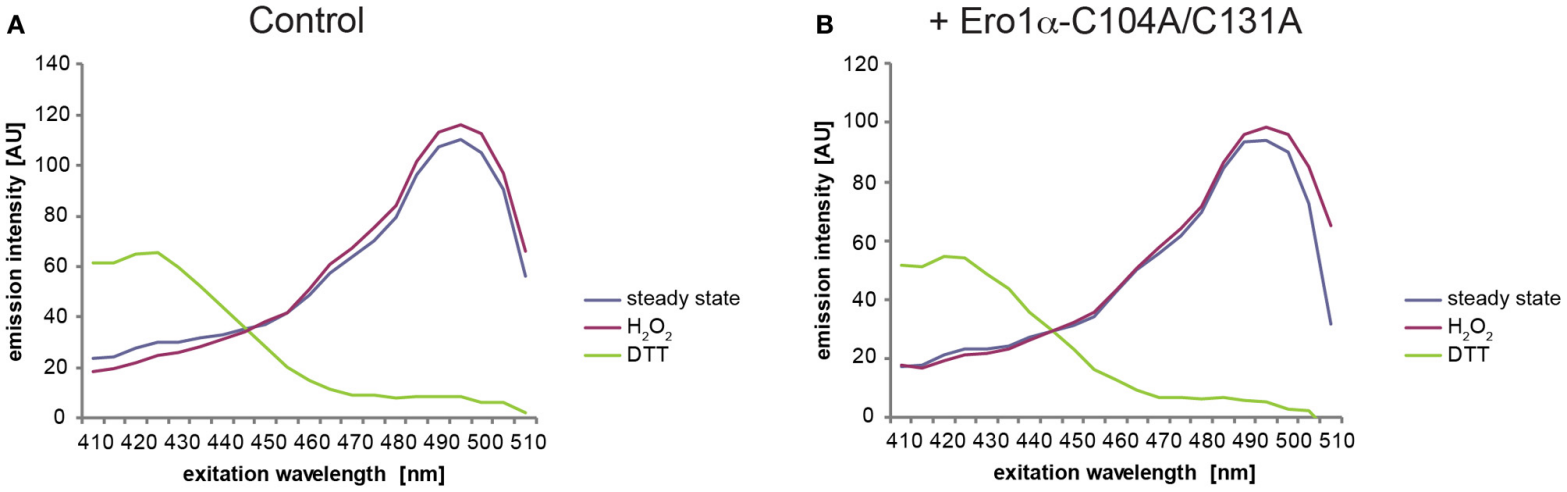
**Steady-state oxidation of HyPer_ER_ changes upon expression of Ero1α-C104A/C131A.** Example spectra from experiments conducted with FlipIn TRex 293:Ero1α-C104A/C131A cells stably transfected with HyPer_ER_. Cells were left untreated **(A)** or Ero1α-C104A/C131A expression was induced for 24 h by addition of 1μg/ml doxycycline **(B)**. The lines in the graphs represent the averaged and blank corrected fluorescence intensities recorded from cells at steady state (blue), upon oxidation with H_2_O_2_ (red) or reduction with DTT (green). The redox-dependent ratiometric behavior of the two peaks of HyPer_ER_ at 420 and 500 nm can readily be appreciated.

Depicted in Figure 6 is a typical outcome of an excitation spectrum analysis of HyPer_ER_ stably expressed in FlipIn TRex 293:Ero1α-C104A/C131A cells. As expected, the steady-state excitation ratio of HyPer_ER_ (blue line) was slightly shifted toward the spectrum obtained from completely oxidized cells (red line) upon expression of Ero1α-C104A/C131A (see Figure 8A for quantification).

**Figure 7:**
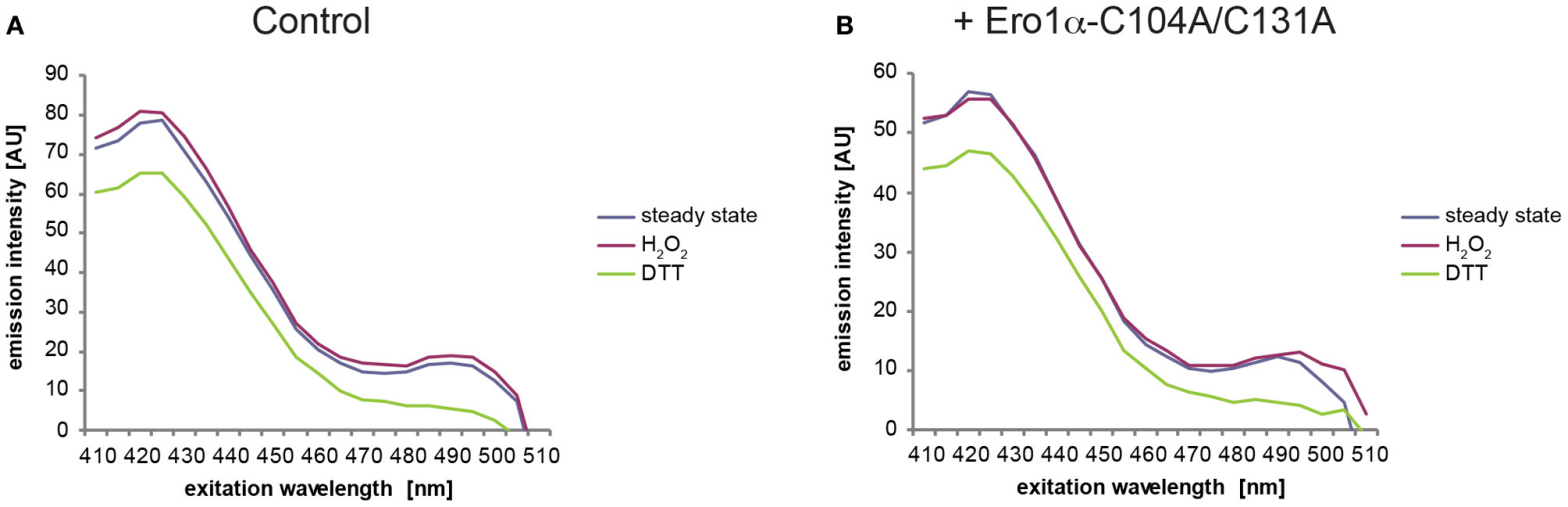
**DTT treatment changes the fluorescence excitation spectrum of SypHer_ER_.** Fluorescence excitation spectra were recorded from uninduced **(A)** or induced **(B)** FlipIn TRex 293:Ero1α-C104A/C131A cells stably transfected with SypHer_ER_ as in Figure 6. Note the lack of redox-dependent ratiometric behavior and the decreased fluorescence intensity in the case of DTT-treated samples.

**Figure 8:**
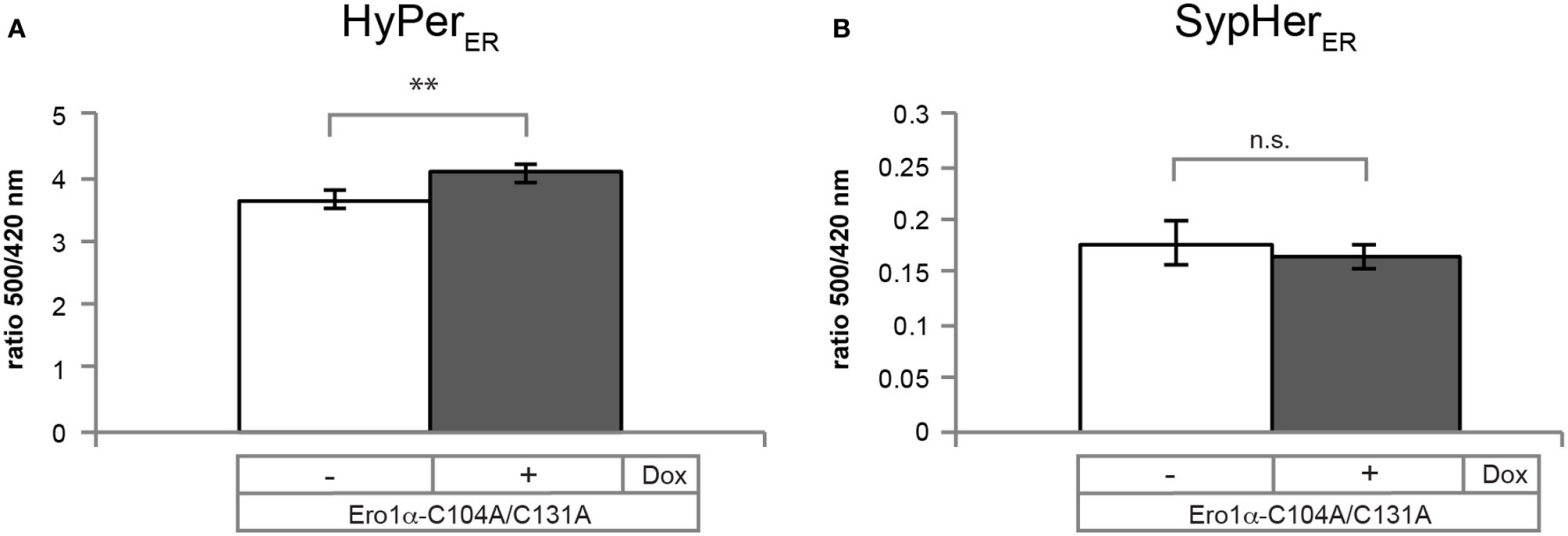
**Ero1α-C104A/C131A-induced changes in the 500/420 nm ratios of HyPer_ER_ and SypHer_ER_.** Plotted are the ratios of the 500 and 420 nm peak amplitudes of the steady state spectra of HyPer_ER_ **(A)** or SypHer_ER_ **(B)**, which were obtained as shown in Figures 6, 7, respectively. Data sets were analyzed for statistical significance using student's *T*-test (two-tailed distribution; heteroscedastic) (*n* ≥ 3; mean ± SD). Dox, doxycycline; ^**^p = 0.009.

Because the fluorescence excitation spectrum of HyPer is not only influenced by reduction/oxidation, but also by changes in pH (Belousov et al., 2006; Forkink et al., 2010; Schwarzlander et al., 2011), appropriate controls are required. To this end, the pH-sensitive C199S mutant of HyPer (termed “SypHer”) (Poburko et al., 2011), which does not respond to redox changes, can be employed. As illustrated in Figure 7, SypHer_ER_ spectra obtained from untreated (blue line) or H_2_O_2_-treated cells (red line) are virtually identical. However, in the case of DTT-treated cells (green line), a decrease in fluorescence emission affecting the entire spectrum is evident. Unfortunately, this effect renders the calculation of OxD of HyPer_ER_ obsolete, at least when employing DTT as a reducing agent. We therefore recommend the use of the HyPer fluorescence intensity ratio upon excitation at 500 nm over excitation at 420 nm of the steady state spectra (blue line), which serves as a robust readout of HyPer_ER_ oxidation (Enyedi et al., 2010; Wu et al., 2010; Malinouski et al., 2011). Analysis of the 500:420 nm fluorescence ratios readily allows the detection of the oxidative shift in the ER elicited by Ero1α-C104A/C131A induction (Figure 8).

## Concluding remarks and future challenges

Redox sensing is a complicated issue. A first layer of complexity is given by the often times short-lived nature of intracellular redox changes, which are typically rapidly reverted by dedicated machinery. For the same reason, oxidative signals are usually locally restricted so that they can be barely detected by global monitoring methods. Because redox-sensitive fluorescent proteins can easily be targeted to subcellular compartments and monitored at real-time resolution, this spatiotemporal problem can now be tackled (Meyer and Dick, 2010). More elaborate targeting of the biosensors to organelle substructures such as membrane contact sites with other cell organelles (Helle et al., 2013) is a future challenge, which will likely lead to a more sophisticated understanding of the context and function of redox changes.

A second layer of complexity in redox monitoring arises from the issue of specificity (or lack of specificity) of the readout. Thus, a sensor protein, which can become oxidized to form a disulfide bond, can in principle react with any other redox couple present so that the OxD of the sensor results from the integration of a variety of oxidizing and reductive inputs. It is important to note that the weight of these different inputs does not reflect their degree of impact on “ER redox conditions” but rather the specific reaction kinetics of the probe with the available redox couples. Based on this principle, quasi-specific probes have been developed by selectively increasing the reaction rate toward a redox couple (e.g., GSH) through intramolecular fusion of a catalyzing enzyme [e.g., glutaredoxin (Meyer and Dick, 2010)]. While such specific readouts certainly constitute a major advance, they also open our view on the wealth of information we do not have yet. For instance, ascorbate–dehydroascorbate (Zito et al., 2012) or reduced–oxidized pyridine nucleotides (Lavery et al., 2008), which at present cannot be measured directly, are other ER redox couples with a reported impact on ER homeostasis.

Finally, it is important to mention that the use of genetically encoded redox biosensors is not restricted to cell culture models, as described herein. Recently, cytosolic and mitochondrial roGFPs sensitive for E_GSH_ or [H_2_O_2_] were expressed and imaged in fruit flies, which led to the stunning conclusion that these two redox systems are surprisingly uncoupled *in vivo* (Albrecht et al., 2011). Clearly, it will also be interesting to determine changes in one or more ER redox couples *in vivo*, both under physiological and pathological situations known to involve the UPR.

### Conflict of interest statement

The authors declare that the research was conducted in the absence of any commercial or financial relationships that could be construed as a potential conflict of interest.
