# Erratum: Green fluorescent protein-based monitoring of endoplasmic reticulum redox poise

**DOI:** 10.3389/fgene.2013.00255

**Published:** 2013-11-26

**Authors:** Christian Appenzeller-Herzog, Julia Birk

**Affiliations:** Division of Molecular and Systems Toxicology, Department of Pharmaceutical Sciences, University of BaselBasel, Switzerland

**Keywords:** endoplasmic reticulum, endoplasmic reticulum stress, glutathione, green fluorescent protein, hydrogen peroxide, unfolded protein response

An error has been identified in the equation to calculate roGFP oxidation (OxD) after our article was published in Frontiers in Genetics. The current equation wrongly features the ratio of ox/red average fluorescence emissions at 390 nm excitation in the denominator. This should be the ox/red ratio of average fluorescence emissions at 465 nm. Accordingly, the correct equation should read:

$${\text{OxD}}_{\text{roGFP}}=\frac{R-R_{\text{red}}}{\frac{I_{465\text{ nm}}\text{ox}}{I_{465\text{ nm}}\text{red}}(R_{\text{ox}}-R)+(R-R_{\text{red}})}$$

